# Comparison of Nonlinear Models for Describing the Growth Curve of Pekin Ducks

**DOI:** 10.1002/vms3.70268

**Published:** 2025-02-14

**Authors:** Navid Ghavi Hossein‐Zadeh

**Affiliations:** ^1^ Department of Animal Science Faculty of Agricultural Sciences University of Guilan Rasht Iran

**Keywords:** absolute growth rate, duck, growth curve, mathematical model

## Abstract

**Background:**

Ducks are used to produce a significant amount of meat in some regions of the world. The enhancement of duck production systems necessitates the development of parameterized mathematical models that accurately depict duck growth, encompassing the assessment of various management and nutritional strategies.

**Objective:**

This research aimed to elucidate the growth trajectory of Danish and French Pekin ducks by applying nonlinear modelling techniques.

**Methods:**

A total of 10 nonlinear mathematical functions (Brody, negative exponential, logistic, Gompertz, Von Bertalanffy, Richards, Schumacher, Morgan, Lomolino and Weibull) were employed in this study. The performance of the models was systematically evaluated, and the behaviour of the models was scrutinized throughout the process of fitting nonlinear regression curves. The overall goodness‐of‐fit for each model to a variety of data profiles was determined through the application of the adjusted coefficient of determination, root mean square error, Akaike's information criterion and Bayesian information criterion.

**Results:**

The adjusted coefficient of determination values associated with each model exhibit generally elevated levels, signifying that the models demonstrate a strong overall compatibility with the data. According to the established goodness‐of‐fit criteria, the Gompertz model emerged as the most suitable mathematical function for accurately representing the growth trajectory of Danish and French Pekin ducks. In contrast, the Brody model exhibited the least favourable fit to the growth patterns of Danish and French Pekin ducks. Analysing the first derivative of the Gompertz model, the absolute growth rates for Danish and French Pekin ducks as a function of time indicated a progressive increase in these rates with advancing age, peaking at 23 and 24 days of age, respectively, before subsequently declining.

**Conclusion:**

An analysis of the various growth models employed in this research revealed that nonlinear functions have the ability to fit Pekin duck body weight records.

## Graphical abstract

The growth patterns of Danish and French Pekin ducks could be adequately described by 10 nonlinear models that were examined in this study. By examining the growth of ducks, the results of this study can facilitate the selection of animals with high productivity and inform strategic management practices. After determining the optimal model for Pekin Duck's growth curve, it is important to suggest the most effective strategies to adapt the growth model parameters to achieve the desired growth curve.

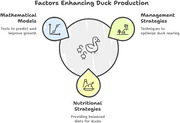

## 1 Introduction

Although poultry meat and eggs are mostly produced by chickens, a considerable amount of meat is produced from ducks in some parts of the world. Waterfowl meat and eggs are highly nutritious foods that are sold for relatively little money (Jalaludeen and Churchil 2022). About 7.5% of global poultry production comes from the production of ducks and geese (Stipkovits and Szathmary 2012; Faridi et al. 2014; El Sabry and Almasri 2023). Poultry‐derived protein sources are highly valuable in human diets. Development of duck‐raising sources and research into the more cost‐effective production of these sources are also necessary in addition to other poultry in order to ensure that human nutrition is adequate (Önk et al. 2018; Hossain et al. 2021; Churchil and Jalaludeen 2022). China was the breeding ground for the Pekin duck, which was brought to Britain in 1872. In Europe and the United States, it has been developed into a number of varieties. In America, the Pekin is the most favoured market duck (Su 2022). It is only bred in the white variety. Orange shanks and toes are preferred, along with creamy white feathers. A black spot on the bill or bean indicates a serious flaw. The bill should be a rich yellow colour. The body can hold a lot of meat because of its long, broad, deep and full breasted shape. A Pekin duck weighs about 8 lb, and a drake weighs 9 lb. Despite being a popular commercial duck, the Pekin is gaining popularity as a pet. Their lifespan is 8–12 years. Ducklings’ feathers are yellow. The Pekin is used for both decorative and meat purposes (Chen et al. 2021; Su 2022).

Growth curves represent changes in growth over time. Growth is defined as changes in live weight and proportionate body component growth influenced by genotype and environmental factors (Camdeviren and Tasdelen 2002; Afrouziyeh et al. 2021). Growth functions allow combining data from recorded measurements into some summary parameters with meaningful interpretation that describe changes in body weight over time (Li et al. 2024). In order to determine the optimal slaughter age and give feeding recommendations, it makes sense to examine the growth performance of an animal in the course of its life (Ghavi Hossein‐Zadeh 2024). The parameters of the growth curve provide a potentially useful basis for selection to change the age–body weight relationship (Kachman and Gianola 1984; Barrera‐Rivera et al. 2024). A favourable growth curve can be attained by choosing growth curve parameters with the right values (Bathaei and Leroy 1998; Purwin et al. 2024). The potential for altering the growth curve's shape through breeding or genetic selection may be of interest to farm animal producers. Direct selection on the growth curve level for individual animals may be possible if the appropriate mathematical model is chosen to represent a growth curve. To attain the intended growth shape, a strategy for modifying the growth model's parameters must be developed (Ghavi Hossein‐Zadeh 2014, 2024; Barrera‐Rivera et al. 2024).

The duck farming industry has grown to be very specialized and well organized. Parameterizing mathematical models of duck growth requires evaluating various management and dietary strategies to optimize duck production systems (Schinckel et al. 2013; Jalaludeen and Churchil 2022; Ghavi Hossein‐Zadeh 2024). However, there are not many studies in which the growth curves of various duck breeds are reported. To incorporate growth functions into the study and production of ducks, this study aimed to determine whether 10 nonlinear models (Brody, negative exponential, logistic, Gompertz, Von Bertalanffy, Richards, Schumacher, Morgan, Lomolino and Weibull) are suitable for describing the growth pattern of Danish and French Pekin duck breeds.

## 2 Materials and Methods

### 2.1 Data Sets

This study employed body weight records for Danish and French Pekin ducks (Kokoszyński et al. 2019). Data from Kokoszyński et al. (2019) have the body weight of the Pekin ducks of lines P8 (Danish Beijing) and P9 (French Beijing), which were part of the Polish program for the preservation of genetic resources, measured at the ages of 1, 7, 14, 21, 28, 35, 42 and 49 days old.

### 2.2 Nonlinear Models

Table 1 displays the nonlinear models that were utilized to explain the growth curves. The following models were fitted to the data to model the relationship between age and body weight: Brody, negative exponential, logistic, Gompertz, Von Bertalanffy, Richards, Schumacher, Morgan, Lomolino and Weibull.

**TABLE 1:** Functional forms of equations used to describe the growth curve of ducks.

Equation	Functional form
Brody	$y=a(1-be^{-kt})$
Negative exponential	$y=a-(ae^{-kt})$
Logistic	$y=\frac{a}{1+be^{-kt}}$
Gompertz	$y=ae^{-be^{-kt}}$
Von Bertalanffy	$y=a{\left(1-be^{-kt}\right)}^{3}$
Richards	$y=\frac{a}{{\left(1-be^{-kt}\right)}^{\frac{1}{m}}}$
Schumacher	$y=\frac{ab^{2}k}{{\left(t+b\right)}^{2}}e^{\left(\frac{bkt}{t+b}\right)}$
Morgan	$y=ab^{k}k\frac{t^{k-1}}{{\left(t^{k}+b^{k}\right)}^{2}}$
Lomolino	$y=\frac{a}{1+b^{\log(\frac{k}{t})}}$
Weibull	$y=a-\left(a-b\right)e^{-\left(\frac{k-1}{k}\right){\left(\frac{t}{IP}\right)}^{k}}$

*Note: y* represents body weight at age t (day); *a* represents asymptotic weight, which is interpreted as mature weight; and *b* is an integration constant related to initial animal weight. The value of *b* is defined by the initial values for *y* and *t*; *k* is the maturation rate, which is interpreted as weight change in relation to mature weight to indicate how fast the animal approaches adult weight; *m* is the parameter that gives shape to the curve by indicating where the inflection point occurs; IP is inflection point.

### 2.3 Statistical Analysis

The NLIN and MODEL procedures in SAS were used to independently fit 10 nonlinear models to duck body weight records to estimate their parameters (SAS Institute 2002). Gauss–Newton method‐based iteration techniques were used to fit nonlinear functions. The NLIN and MODEL procedures assess the initial parameter value specifications before this process is started. The growth curve of the ducks was fitted with the sinusoidal function using SigmaPlot 15.0's nonlinear regression technique (Systat Software Inc., San Jose, CA, USA). The Marquardt–Levenberg algorithm was used iteratively to estimate the model parameters. For the iterative process to work, the initial values of the parameters had to be specified. The final estimates were not influenced by the initial values chosen.

The models were assessed for their goodness‐of‐fit or predictive accuracy using various statistical metrics, including the adjusted coefficient of determination ($R_{\mathrm{adj}}^{2}$), residual standard deviation, root mean square error (RMSE), Durbin–Watson (DW) statistic, Akaike's information criterion (AIC) and Bayesian information criterion (BIC).

The formula for calculating $R_{\mathrm{adj}}^{2}$ was as follows:

$$R_{\mathrm{adj}}^{2}=1-\left[\frac{\left(n-1\right)}{\left(n-p\right)}\right]\left(1-R^{2}\right)$$

In this context, $R^{2}$($R^{2}=1-\frac{\mathrm{RSS}}{\mathrm{TSS}}$) represents the multiple coefficient of determination, *n* indicates the number of observations (data points) and *p* refers to the number of parameters. Additionally, RSS denotes the residual sum of squares, whereas TSS represents the total sum of squares. The $R^{2}$ value ranges between 0 and 1, with higher values (close to 1) indicating a more satisfactory model fit.

RMSE is computed using the following formula:

$$\mathrm{RMSE}=\frac{\mathrm{RSS}}{\sqrt{n-p-1}}$$

The RMSE plays a vital role in assessing the suitability of growth curve models, with the most appropriate model being the one that yields the lowest RMSE.

To detect autocorrelation in the residuals from the regression analysis, the DW statistic was employed. The DW statistic has a range of 0–4: A value near 2 indicates no autocorrelation, a value close to 0 suggests positive autocorrelation, and a value near 4 implies negative autocorrelation (Ghavi Hossein‐Zadeh 2014). The following formula was used to determine the DW statistic:

$$\mathrm{DW}=\frac{\sum_{t}^{n}{\left(e_{t}-e_{t-1}\right)}^{2}}{\sum_{t=1}^{n}e_{t}^{2}}$$

where $e_{t}$ denotes the residual at time *e*, and $e_{t-1}$ presents residual at time *t* − 1.

AIC was calculated as follows (Burnham and Anderson 2002):

$$\mathrm{AIC}=n\times \ln\left(\mathrm{RSS}\right)+2p$$

The AIC adjusts the RSS based on the number of parameters in the model. In model comparisons, a lower AIC value indicates a superior fit.

In contrast, the BIC penalizes the (log) maximum likelihood with a term that takes model complexity into account, thereby integrating maximum likelihood (data fitting) with choice of model:

$$\mathrm{BIC}=n\ln\left(\frac{\mathrm{RSS}}{n}\right)+p\ln\left(n\right)$$

In the model comparison, a lower BIC value shows a better fit.

Following the identification of the best fitting function, the absolute growth rate (AGR) was computed using its first derivative with respect to time ($\frac{\partial y}{\partial t}$). Specifically, in this context, the AGR corresponds to the estimated daily weight gain throughout the growth period (Ghavi Hossein‐Zadeh 2015).

## 3 Results

For the Danish and French Pekin ducks, the estimated parameters for the nonlinear growth models are displayed in Table 2. Furthermore, Table 3 displays the goodness‐of‐fit statistics for 10 growth models that were fitted to the ducks’ body weight data. All models provided high $R_{\mathrm{adj}}^{2}$ values, and although there were minimal differences in $R_{\mathrm{adj}}^{2}$ values between the models, the Gompertz model had the highest $R_{\mathrm{adj}}^{2}$ value for Danish and French Pekin ducks. Nevertheless, for both duck breeds, the Brody model yielded the lowest values of $R_{\mathrm{adj}}^{2}$. According to DW values for the models fitted on body weight records of both duck breeds, positive autocorrelation was observed for the Brody and negative exponential models, but negative autocorrelation for the Gompertz, Richards, Scumacher and Weibull models. Moreover, the logistic, Von Bertalanffy, Morgan and Lomolino models did not show any autocorrelation. For Danish and French Pekin ducks, the Gompertz and Brody models provided the lowest and highest RMSE, AIC and BIC values, respectively. The best function for fitting the growth curve of both duck breeds was therefore found as a Gompertz model. It was also found that the Brody model corresponds to the worst of the duck growth curve.

**TABLE 2:** Parameter estimates (standard errors are in parentheses) for the different growth models in ducks.

		Model									
Breed	Parameter	Brody	Negative exponential	Logistic	Gompertz	Von Bertalanffy	Richards	Schumacher	Morgan	Lomolino	Weibull
	*y*_0_	—	—	—	—	—	—	—	—	—	—
Danish	*a*	−14,015.1	−4987.92	2230.02	2603.78	2974.50	2472.33	41.79	234,354.8	3367.4	2377.6
	*b*	0.9939	—	23.4414	4.3467	0.8610	−1.2733	20.2632	80.16505	95.2484	64.5174
	*k*	−0.00311	−0.00752	0.119732	0.06277	0.04335	0.07484	0.495034	2.63234	36.9441	2.0503
	*m*	—	—	—	—	—	0.20869	—	—	—	—
	IP	—	—	—	—	—	—	—	—	—	24.05
	*y*_0_	—	—	—	—	—	—	—	—	—	—
French	*A*	−11,526.6	−5291.76	2111.62	2511.22	2932.57	2401.12	82.91	264,080.8	3683.9	2327.4
	*b*	0.9950	—	20.2639	4.0042	0.8093	−0.7805	23.6164	89.98532	55.6420	68.7798
	*k*	−0.00344	−0.0067	0.11262	0.057903	0.039018	0.06647	0.398277	2.514388	44.3791	1.9212
	*m*	—	—	—	—	—	0.153525	—	—	—	—
	IP	—	—	—	—	—	—	—	—	—	24.07

**TABLE 3:** Comparing goodness of fit for different growth curves in ducks.

		Model									
Breed	Statistics	Brody	Negative exponential	Logistic	Gompertz	Von Bertalanffy	Richards	Schumacher	Morgan	Lomolino	Weibull
	$R_{\mathrm{adj}}^{2}$	0.9822	0.9825	0.9957	0.9976	0.9960	0.9973	0.9970	0.9963	0.9954	0.9969
	DW	1.50	1.30	2.23	3.20	2.43	3.37	2.81	2.39	2.13	3.31
Danish	RMSE	106.6	105.7	52.67	39.17	50.40	41.62	43.95	48.79	54.25	44.30
	AIC	93.58	92.90	82.30	77.56	81.60	78.75	79.40	81.08	82.77	79.75
	BIC	77.19	76.42	65.90	61.16	65.20	62.43	63.01	64.68	69.27	67.29
	$R_{\mathrm{adj}}^{2}$	0.9871	0.9878	0.9965	0.9986	0.9974	0.9984	0.9981	0.9970	0.9959	0.9980
	DW	1.47	1.32	1.86	3.31	2.39	3.41	2.85	1.93	1.79	3.29
French	RMSE	83.14	80.79	43.47	27.58	37.45	29.60	31.68	39.95	46.91	32.97
	AIC	89.60	88.60	79.23	71.95	76.84	73.29	74.17	77.88	80.45	75.02
	BIC	73.21	72.13	62.83	55.55	60.45	56.98	57.77	61.49	66.95	62.56

**FIGURE 1:**
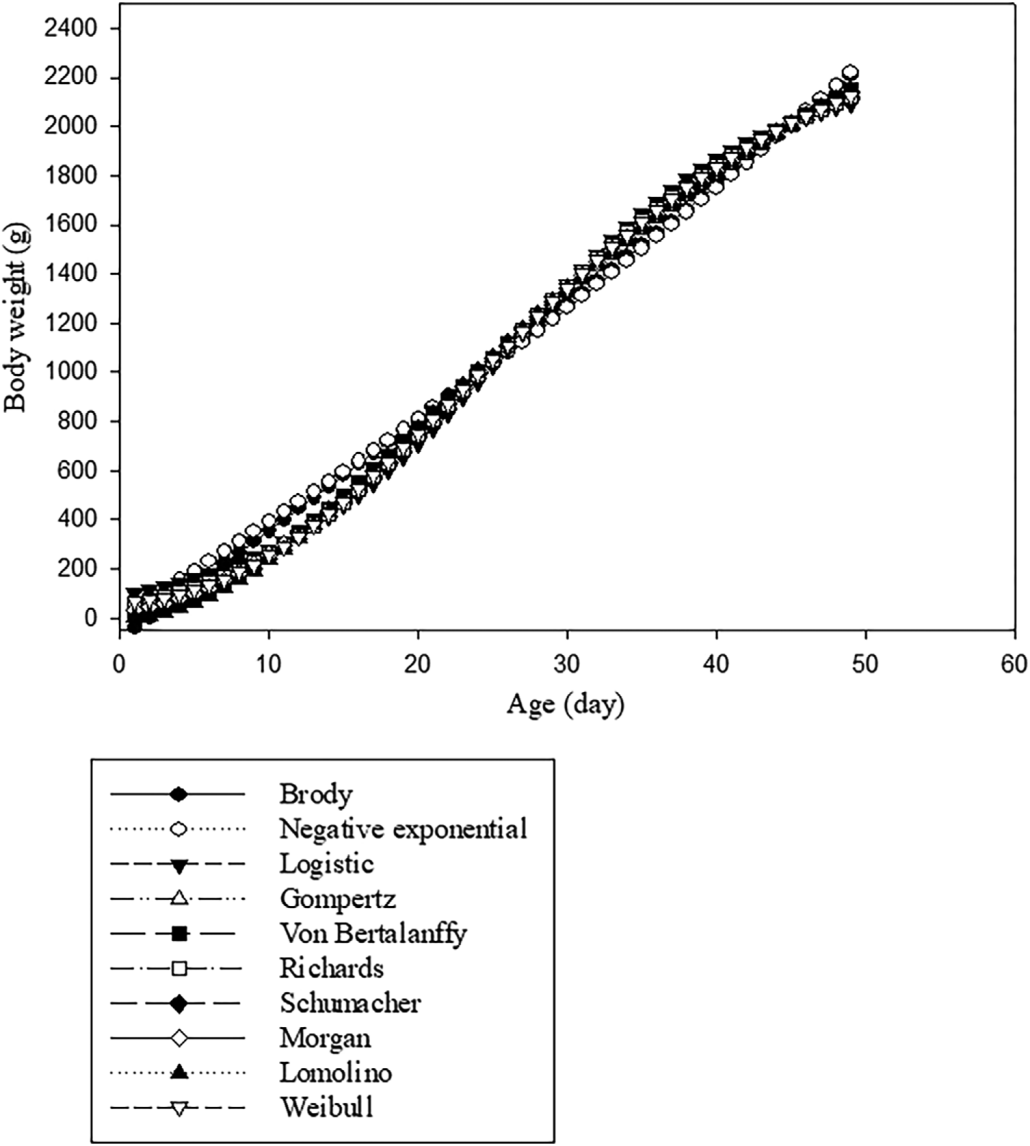
Predicted body weights as a function of age, determined using different nonlinear models for Danish Pekin ducks.

**FIGURE 2:**
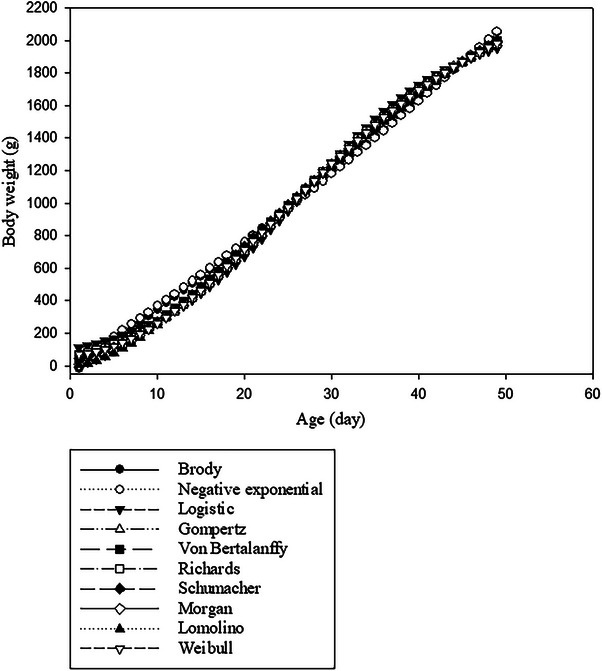
Predicted body weights as a function of age, determined using different nonlinear models for French Pekin ducks.

According to Kokoszyński et al. (2019), body weights of Danish and French Pekin ducks were found to increase with age during the study period, with Danish Pekin ducks becoming noticeably heavier than French Pekin ducks after 49 days of age. The Brody model did not provide a suitable prediction from initial body weight. For Danish Pekin ducks, the negative exponential, Von Bertalanffy, Gompertz, Schumacher, Lomolino and Morgan functions provided underestimated initial body weights. Moreover, the logistic and Weibull models overestimated the initial body weights. However, the Richards model predicted initial body weights close to the actual value in Danish Pekin ducks. For French Pekin ducks, the negative exponential, Von Bertalanffy, Schumacher, Lomolino and Morgan functions provided underestimated initial body weights. Moreover, the logistic, Richards and Weibull models overestimated the initial body weights. However, the Gompertz model predicted initial body weights close to the actual value in French Pekin ducks. The predicted final or asymptotic body weights varied in magnitude across the different functions. Although the Brody and negative exponential models overestimated the final body weights in Danish Pekin ducks, the other models demonstrated accurate predictions. In contrast, all models effectively predicted the final body weight for French Pekin ducks. On the basis of different nonlinear models, the predicted body weights for Danish and French Pekin ducks as a function of age are shown in Figures 1 and 2, respectively.

**FIGURE 3:**
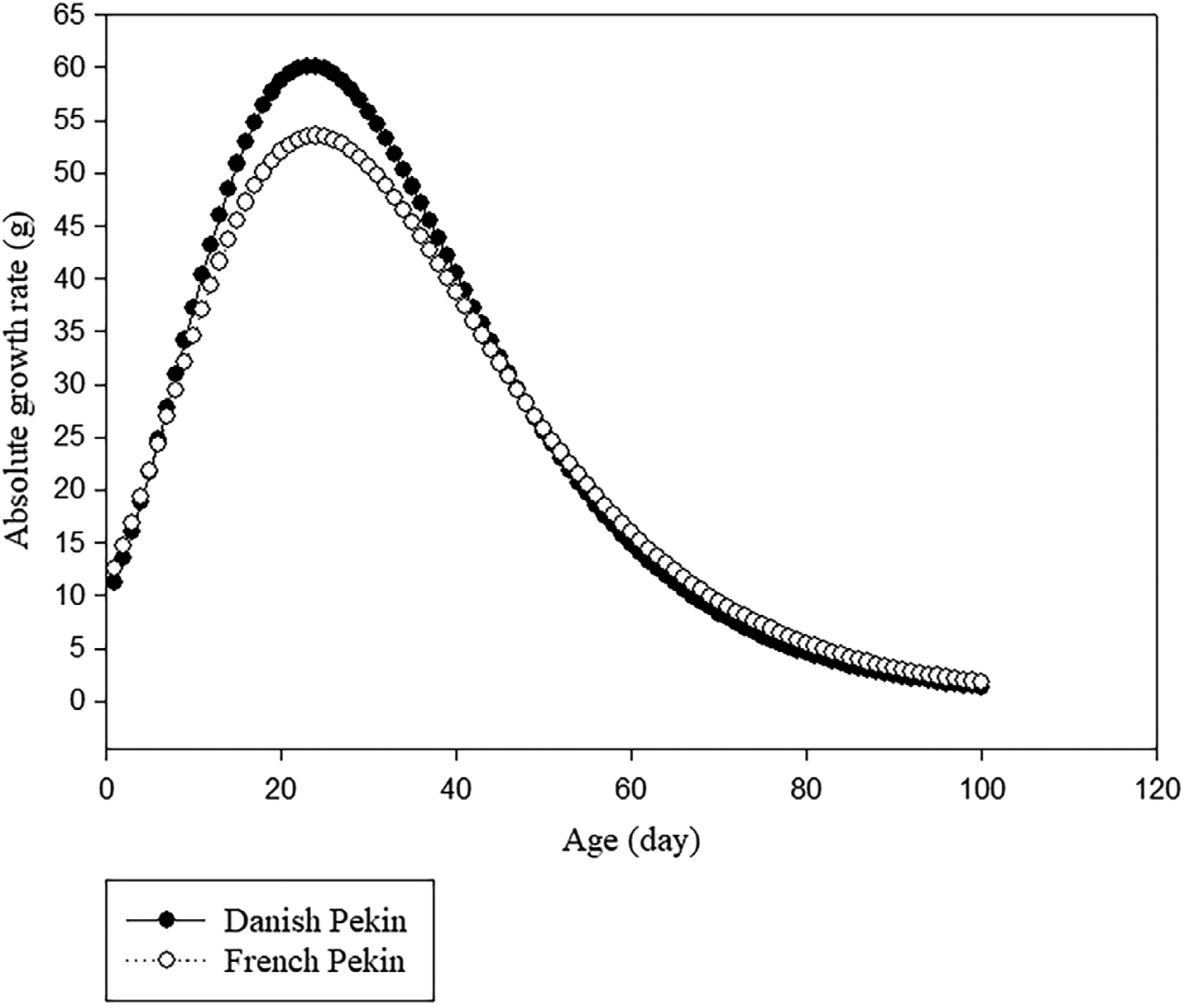
Absolute growth rate (AGR) for Danish and French Pekin ducks based on the Gompertz model.

Figure 3 depicts the AGR values for Danish and French Pekin ducks over time, derived from the first derivative of the Gompertz model. For both Danish and French Pekin ducks, AGR values showed a gradual increase with age, peaking at 23 and 24 days of age, respectively, before beginning to decline.

## 4 Discussion

Fitting growth curves and analysing their parameters is a fundamental task for the breeding and production of livestock and poultry. The most appropriate model to explain live weight data for Pekin ducks was found in this study. The right feeding strategy and animal selection are determined by calculating the growth curve parameters, which also provide an accurate description of topics like growth, animal performance and the ideal age at slaughter. A growth curve's level can be the exclusive focus of selection after an appropriate growth curve model has been chosen. For the growth model to take on the desired growth shape, the optimal course of action for changing its parameters must be determined.

Nonlinear growth models are helpful in accurately characterizing the growth trajectories of poultry species, including Pekin ducks. Each model offers unique parameters and flexibility, allowing for the capture of various growth dynamics. The models selected for this study (Brody, negative exponential, logistic, Gompertz, Von Bertalanffy, Richards, Schumacher, Morgan, Lomolino and Weibull) have been widely utilized in poultry growth research (Haqani et al. 2021; Machado et al. 2023; Ghavi Hossein‐Zadeh 2024; Osaiyuwu et al. 2024; Şengül et al. 2024; Ussyarif, Kurnianto, and Setiaji 2024). The selection of these models was intended to explore a comprehensive range of growth dynamics, from simple to complex, ensuring a thorough evaluation of potential growth patterns in Pekin ducks. By using this diverse model set, the most suitable function should be determined, which precisely records the growth curve of Danish and French Pekin ducks and thus provides valuable findings for the optimization of their production systems. Therefore, all functions analysed in this study demonstrated a strong alignment with the data sources of Pekin ducks, as determined by assessing their behaviour and statistical performance characteristics. In general, some notable variations in the characteristics that indicate Pekin duck growth in the future were found when evaluating the growth models using statistical criteria. The Gompertz growth function was identified as the most effective in explaining age‐related changes in the body weight of Pekin ducks based on goodness‐of‐fit criteria. In order to select the best model, however, it must be carefully examined how animals develop in different surrounding environments (Dogan et al. 2010). This is particularly important in cases in which data are collected under previously unidentified circumstances, as with certain poultry species or ducks that grew up in unusual environments or climate zones (Darmani Kuhi et al. 2019).

The growth curves of different duck breeds have been studied by researchers using nonlinear models. Consistent with the findings of this study, Önk et al. (2018) and Thinh et al. (2021) determined that the Gompertz model was best suited for modelling the growth curves of native ducks raised under different systems in Turkey and eastern spotted‐billed ducks in Vietnam, respectively. Conversely, Anang et al. (2017) and Almeida et al. (2018) concluded that the logistic model was most appropriate for representing the growth patterns of Rambon ducks and native duck breeds. Additionally, Asiamah et al. (2020) observed that the logistic, Gompertz and Von Bertalanffy models exhibited similar levels of accuracy in describing the growth patterns of male and female Leizhou black ducks, with the Von Bertalanffy model emerging as the most accurate overall.

Although the Gompertz function had a fixed inflection point, it performed well in predicting the growth behaviour of both duck breeds in this study. Other advantages of the Gompertz model were the easy convergence and the simple biological interpretation of the parameters. The significant differences in the fit of growth functions across duck breeds can be attributed to variations in their growth curve characteristics. Body weight records, breed characteristics, the amount of data points and the mathematical structure of the models can cause all variations of model adaptation that are observed across studies. Growth curve variations are influenced by both environmental and genetic factors.

On the basis of the DW statistic values obtained from fitting nonlinear growth curve models in this study, it was concluded that some models exhibited both positive and negative autocorrelations in the residuals, whereas others showed no autocorrelation. This suggests that issues related to residual autocorrelation were minimal or absent in the analysis. Serial correlation, also called positive autocorrelation, is the phenomenon in which a positive error in one observation increases the probability of a positive error in another (Ghavi Hossein‐Zadeh 2014).

The AGR values reflect the relative importance of an animal's growth throughout its life. AGR represents the average weight gain of animals per unit of time along the growth trajectory. The results revealed that the AGR for both duck breeds reached its peak at the inflection point, where growth rate was at its maximum. Beyond this point, the AGR began to decline steadily, eventually reaching minimal values at the end of the analysis period, indicating no further weight gain. It is possible that poor management practices are the cause of this AGR decline. To enhance weight gain during this critical phase, adjustments to feed management are necessary.

## 5 Conclusion

The growth patterns of Danish and French Pekin ducks could be adequately described by the ten nonlinear models that were examined in this study. For both breeds, the Gompertz model demonstrated the most favourable fit for growth curves, as evidenced by the comparatively lower values of RMSE, AIC and BIC, along with elevated values of $R_{\mathrm{adj}}^{2}$ in contrast to alternative models. By scrutinizing the growth trajectories of the animals, the outcomes of the study can facilitate the selection of animals with high productivity and inform strategic farm management practices. After determining the optimal model for the growth curve of Pekin ducks, it is crucial to propose the most effective strategies for adjusting the parameters of the growth model to attain the desired growth curve.

## Author Contributions

**Navid Ghavi Hossein‐Zadeh**: writing–review and editing, writing–original draft, validation, supervision, project administration, investigation, conceptualization.

## Ethics Statement

The author has nothing to report.

## Conflicts of Interest

The author declares no conflicts of interest.

### Peer Review

The peer review history for this article is available at https://publons.com/publon/10.1002/vms3.70268.

## Data Availability

No data are associated with this manuscript.
